# Genetic Determinants of the Acute Respiratory Distress Syndrome

**DOI:** 10.3390/jcm12113713

**Published:** 2023-05-27

**Authors:** Eva Suarez-Pajes, Eva Tosco-Herrera, Melody Ramirez-Falcon, Silvia Gonzalez-Barbuzano, Tamara Hernandez-Beeftink, Beatriz Guillen-Guio, Jesús Villar, Carlos Flores

**Affiliations:** 1 Research Unit, Hospital Universitario Nuestra Señora de Candelaria, 38010 Santa Cruz de Tenerife, Spain; 2 Department of Population Health Sciences, University of Leicester, Leicester LE1 7RH, UK; 3 NIHR Leicester Biomedical Research Centre, University of Leicester, Leicester LE1 7RH, UK; 4 CIBER de Enfermedades Respiratorias (CIBERES), Instituto de Salud Carlos III, 28029 Madrid, Spain; 5 Research Unit, Hospital Universitario de Gran Canaria Dr. Negrín, 35019 Las Palmas de Gran Canaria, Spain; 6 Genomics Division, Instituto Tecnológico y de Energías Renovables (ITER), 38600 Santa Cruz de Tenerife, Spain; 7 Faculty of Health Sciences, University of Fernando Pessoa Canarias, 35450 Las Palmas de Gran Canaria, Spain

**Keywords:** ARDS, genomics, biomarkers, rare genetic variation, GWAS, exome, COVID-19

## Abstract

Acute respiratory distress syndrome (ARDS) is a life-threatening lung condition that arises from multiple causes, including sepsis, pneumonia, trauma, and severe coronavirus disease 2019 (COVID-19). Given the heterogeneity of causes and the lack of specific therapeutic options, it is crucial to understand the genetic and molecular mechanisms that underlie this condition. The identification of genetic risks and pharmacogenetic loci, which are involved in determining drug responses, could help enhance early patient diagnosis, assist in risk stratification of patients, and reveal novel targets for pharmacological interventions, including possibilities for drug repositioning. Here, we highlight the basis and importance of the most common genetic approaches to understanding the pathogenesis of ARDS and its critical triggers. We summarize the findings of screening common genetic variation via genome-wide association studies and analyses based on other approaches, such as polygenic risk scores, multi-trait analyses, or Mendelian randomization studies. We also provide an overview of results from rare genetic variation studies using Next-Generation Sequencing techniques and their links with inborn errors of immunity. Lastly, we discuss the genetic overlap between severe COVID-19 and ARDS by other causes.

## 1. Introduction

Acute respiratory distress syndrome (ARDS) develops by an injury to the capillary alveolar membrane triggered by direct or indirect causes, including sepsis, severe pneumonia, major trauma, acute pancreatitis, or multiple blood transfusion, among others [1,2]. While symptoms vary widely, all patients require hospitalization in the Intensive Care Unit (ICU) due to a range of serious complications [3,4]. ARDS patients are characterized by non-cardiogenic pulmonary protein-enriched edema and bilateral infiltration, detectable by chest imaging [5]. Lung damage affects gas exchange, leading to hypoxemia and acute respiratory failure.

ARDS diagnosis is based on clinical and imaging criteria, which have been updated over the years from the previous AECC (American-European Consensus Conference) definition [6,7] to the current Berlin definition released in 2012 [8]. The lack of specific diagnostic tests has a negative impact on the patients, given that early administration of treatments could improve their prognosis [9,10,11]. In addition, the mortality due to ARDS in the ICUs is still over 40% [12]. Seeking effective treatments and specific prognostic methods becomes a priority to improve the survival of ARDS patients. This situation has been exacerbated by the global pandemic of the coronavirus disease 2019 (COVID-19) caused by SARS-CoV-2. In pre-vaccination periods, the available estimates support that 40% of hospitalized patients and 75% of ICU patients with COVID-19 could have developed ARDS [13].

The current management of ARDS patients is based on life-supporting treatments [14] towards maintaining gas exchange and reducing the acute inflammatory response. Applying lung protective ventilation, prone positioning, and corticosteroid administration are known procedures for improving patient survival [5]. Despite decades of research, specific lung-directed pharmacological treatments with demonstrated benefits in clinical trials are lacking [15,16]. In this context, since the efficiency of clinical trials improve when the drug targets have genetic support [17,18], leveraging genomic information could hold the key to guiding the search for future ARDS treatments.

Nevertheless, ARDS is a complex syndrome that cannot be explained by a single cause. For instance, not all patients diagnosed with COVID-19 or with bacterial pneumonia develop ARDS. Thus, advances in the accurate characterization of patients with sepsis (by any cause) and of other critical conditions triggering ARDS will help to improve risk predictions and move towards better therapies. Since genetic host factors play an essential role in patient predisposition, many genetic studies have aimed to assess the influence of genetics on ARDS severity and mortality [19,20]. However, the high heterogeneity of the patients with the syndrome and the relatively low incidence of ARDS complicate these studies.

This review aims to emphasize the importance of the studies that aim to detect genetic variants and genes associated with increased susceptibility to critical diseases. We also expose alternative state-of-the-art genomic approaches that are becoming key to identifying novel therapeutic targets and biomarkers of ARDS.

## 2. Common Genetic Variation and ARDS Risk and Outcomes

**Figure 1:**
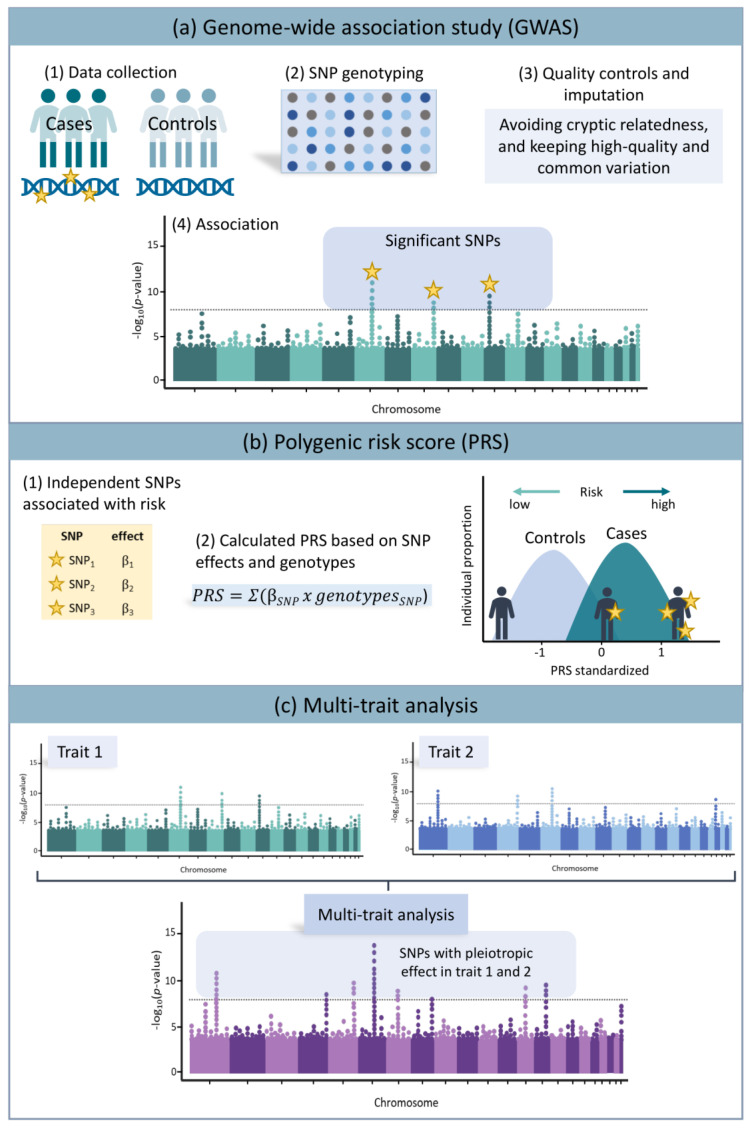
Different approaches based on the analysis of common genetic variants. (**a**) Genome-wide association studies (GWAS) allow screening for common variants across the genome to identify those that are significantly associated with a disease or trait. (**b**) By aggregating the effect of the detected genetic risk variants, one can obtain a simplified measure in the form of a polygenic risk score (PRS), where patients at risk will have higher PRS values compared to controls. (**c**) Using multi-trait analysis, the GWAS results obtained for two or more correlated traits can be used to identify shared genetic loci that converge with effects in a function. Abbreviations: SNP: single nucleotide polymorphism.

Genetic studies in ARDS have evolved from studies focusing on candidate genes to studies relying on hypothesis-free approaches to identify novel loci associated with ARDS risk or severity [20,21]. This has been possible by diverse technical, statistical, and bioinformatic improvements over the last 15 years that facilitate the use of commercial microarrays for efficient low-cost single nucleotide polymorphism (SNP) genotyping and **variant imputation** to conduct **genome-wide association studies** (GWAS) [22]. These studies have allowed the assessment of hundreds of thousands of common genetic variants across the genome and aim to identify disease loci without necessitating previous knowledge of the disease pathogenesis. The development of public resources, such as the TopMed server at https://topmed.nhlbi.nih.gov (accessed on 16 February 2023), has permitted a boost in genome coverage and study power (Figure 1a). To better explain these concepts, a definition table is included (Table 1).

Despite the success of GWAS, its use to disentangle ARDS genetics is yet scarce. In addition, they have been typically conducted assuming additive inheritance models without assessing other inheritance models that depart from additivity. The first GWAS of ARDS was conducted by Christie and colleagues [23], focusing on trauma-induced ARDS. They revealed interest in variants near the *PPFIA1* gene, which encodes liprin-α-1 that is involved in cell adhesion and cell-matrix interactions [23]. A subsequent multi-ancestry GWAS and functional evidence through mouse models supported the selectin P ligand (*SELPLG*) as a novel ARDS gene involved in susceptibility [24]. Intriguingly, elevated levels of the soluble form of P-selectin protein, with a role in platelet activation, have been associated with acute lung injury and have been recently proposed as an indicator of severe forms of COVID-19 [25,26]. The next published GWAS in the timeline was conducted by Guillen-Guio and colleagues [27] on European patients with sepsis-induced ARDS. This study supported that variants in Fms Related Receptor Tyrosine Kinase 1 (*FLT1*) gene, which were involved in gene-expression regulation, were associated with ARDS risk [27]. The encoded protein, VEGFR-1, is a key member of the vascular endothelial complex growth factor (VEGF) pathway, a usual suspect in ARDS by its role in angiogenesis and vascular permeability [28,29,30]. The last study in the list is a GWAS performed by Du and colleagues [31] in a multi-ancestry cohort. This GWAS linked ARDS risk to an SNP intergenic to the BLOC-1 Related Complex Subunit 5 (*BORCS5*) and Dual Specificity Phosphatase 16 (*DUSP16*) genes among European patients, and was found to be associated with immune regulation [31]. This association was not replicated in the African American patients of the cohort.

## 3. The Importance of the Pathogen and Large-Scale GWAS: COVID-19 as an Example

The immune response triggered during an infection depends largely on the features of the pathogen, the virulence, the route of infection, and tropism. Multiple microorganisms are relevant in ARDS studies, including bacteria such as *Streptococcus pneumoniae* or *Staphylococcus aureus* and viruses such as influenza, MERS-CoV, SARS-CoV, or SARS-CoV-2, among others [32,33]. However, susceptibility and severity of ARDS segregated by or conditioned by host genetics are not under discussion, especially after seeing the scientific advances unfolded during these three years of the COVID-19 pandemic.

The impact of COVID-19 on global health systems has led to an unprecedented international effort and the establishment of large consortia to characterize the new infectious agent and to determine the genetic host factors that contribute to the variability in symptom presentation. Despite the challenge, international efforts have led to major advances in a short period of time that allowed a rapid advance in the understanding of the disease and the genetics behind the severe forms of the disease. They have also allowed a deep characterization of the virus, in part because many countries deployed genomic surveillance programs that were essential to track the spread and evolution of the virus and for guiding effective treatments. Some of these initiatives have been aimed at large-scale GWAS involving nationwide cohorts or multi-country studies [34,35,36]. While a full list of gene discoveries in COVID-19 is beyond this review, the GWAS performed under The COVID-19 Host Genetic Initiative at https://www.COVID19hg.org (accessed on 16 February 2023), which included genetic data from 219,692 cases and over 3 million controls, have enabled identifying genes involved in the viral entry (e.g., *ACE2*), defense in the airway mucus (e.g., *MUC5B*), and the type I interferon response (e.g., *IFNAR2*) [35]. Among the most robust associations is the *ABO* locus, which has been significantly associated with susceptibility, severity, and respiratory failure [34,36,37]. *ABO* is also involved in blood group determination, and blood type A has been associated with increased infection susceptibility and risk of severe outcomes [38]. In addition, other COVID-19 genes relate to gas exchange and surfactant protein metabolism. This is the case of the association near the *NAPSA* gene, which encodes a protease that is highly expressed in alveolar cells [34,39]. Other studies linked severe COVID-19 with genes involved in edema formation, one of the hallmarks of ARDS. This is the case of the *AQP3* gene [35] that encodes a transmembrane protein involved in water transport [40]. Upregulation of APQ3 has been observed in diffuse alveolar damage induced by different causes [41] and is involved in water transport during pulmonary edema formation [42]. These studies illustrate the role of common host genetic factors in the response to an infection leading to an increased risk of patient hospitalization and a worse prognosis.

The biological processes involved in severe COVID-19 are old suspects for ARDS by other causes. The above-mentioned studies describe genes involved in immune regulation, pulmonary edema formation, or lung protection. However, it is still unknown whether these and other COVID-19-associated genes could also be involved in the genetic risk of ARDS by other causes. The available GWAS results provide the basis for estimating genetic correlations between diseases using linkage disequilibrium (LD) score regressions and the known common disease loci. Studies of genetic overlap have been conducted between COVID-19 and diseases such as idiopathic pulmonary fibrosis, type II diabetes, coronary artery disease, hypertension, or obesity, to name a few [43,44,45]. Extending these approaches to non-COVID-19 ARDS likely could allow the identification of genetic similarities and uncover novel loci of interest.

Large-scale GWAS, such as those available for COVID-19, highlight the importance of international collaborations to retain the study power to detect genetic risk variants in homogeneous groups of patients sharing clinical or molecular characteristics (endotypes). Extending this approach to other infectious diseases and taking into account the interaction between the pathogen and host genetics, as has been performed for *Streptococcus pneumoniae* [46] or *Mycobacterium tuberculosis* [47], could be key to further understanding inter-individual variability in disease progression and severity. We expect that the development of larger GWAS in ARDS, including a focus on both specific or wider causes of ARDS, will provide novel possibilities for further discoveries.

## 4. Beyond GWAS: Polygenic Risk Scores and Multi-Trait Analyses

So far, GWAS has identified a few ARDS genes that may serve as clinical biomarkers or that have fostered further research to demonstrate the utility of novel therapies [23,24,27]. However, common genetic variants typically offer limited power to predict complex conditions such as ARDS. This is because complex traits are typically associated with genetic variants with small effects, and because disease susceptibility is conferred by aggregated effects of thousands of common variants across the genome [48].

The aggregated effects of risk variants can provide an individual estimate of the risk of developing a certain disease or syndrome, and this can be modeled as **polygenic risk scores** (PRSs) or polygenic scores (PGSs), as a more general term for all types of complex traits (Figure 1b) [49,50]. PRS models hold the promise of providing a valuable tool for diverse applications for **Personalized Medicine** [51,52], including early diagnosis, patient risk stratification, prediction of outcomes, and guiding the treatment, to name a few. For instance, PRS models have been used to assess early genetic risk prediction in diverse respiratory diseases, such as pneumonia [53], idiopathic pulmonary fibrosis [54], pulmonary tuberculosis [55], asthma [56], and COVID-19 [35,57,58]. In addition, others have found that severe COVID-19 PRS can distinguish patients with non-COVID-19 ARDS from at-risk controls [31]. Thus, the development of effective PRS models for the diagnosis of ARDS for the identification of at-risk patients could lead to improved treatments and novel models of patient management in the ICU.

For research, among many other applications, PRS models can also be used to assess the degree of genetic overlap between two or more diseases or traits to further understand the underlying biology of the conditions being compared, inform drug repurposing [59], or prioritize patients for carrier gene screening of disease-causing variants with large effects in disease [60,61]. Due to their potential, some studies have been conducted recently, leveraging the possibilities of assessing PRS models to evaluate the genetic overlap of conditions among sepsis patients [62,63]. As an illustrative example, D’Urso and colleagues generated PRS models based on different traits, such as the granulocyte count or C-reactive protein levels, which were subsequently correlated with the presence of septic shock and mortality in patients with sepsis [63].

A complementary genetic approach to identify the overlapping pathogenic mechanisms across diseases is the **multi-trait analysis**. Recently, multi-trait approaches have been developed to rely on GWAS findings of correlated traits to aggregate the effect of multiple associations and improve the understanding of the identified loci that share biological functions (pleiotropy) [64]. Multi-trait analyses increase the statistical power for identifying genetic associations on correlated complex traits [64,65] (Figure 1c). For instance, this approach revealed shared genetic loci involved in severe COVID-19, systemic lupus erythematosus, and rheumatoid arthritis, identifying a strong common locus at the human leukocyte antigen (HLA) region [66]. Genes located at this locus have been extensively linked to immune response and autoimmune diseases, which reinforces the implication of autoimmunity and immune dysregulation during the course of SARS-CoV-2 infection. In addition, other genes not previously linked to COVID-19 were identified.

Increasingly, studies leverage the GWAS results to determine the correlation between diseases and reveal pleiotropic loci. Applying such approaches in the future will help to identify novel ARDS risk genes that could reveal key pathogenic mechanisms which may provide a robust source of targets for pharmacological repositioning [67].

## 5. Genetic Determinants of ARDS Biomarkers

**Biomarkers** can provide insights into the host response to infections, severity, prognosis, and outcomes. Their use in ARDS could be key to providing information for patient risk stratification, for assistance in the diagnosis, and may also be useful in better defining the treatment or its dosage. Several protein biomarkers of ARDS have been described in the literature, including proteins involved in epithelial damage (RAGE, SP-D, and CC16), endothelial permeability (ANG-1, ANG-2, and VEGF), inflammation (IL-6, IL-8, and IL-1RA), and coagulation and fibrinolysis (FiB, PAI-1, and thrombomodulin), among others [68,69]. Despite some studies proposing that a biomarker combination could suffice for disease prediction [70,71], current biomarkers are far from offering a precise prediction of ARDS and are not yet used in clinical practice. Further characterization of biomarkers and their involvement in ARDS is needed.

**Figure 2:**
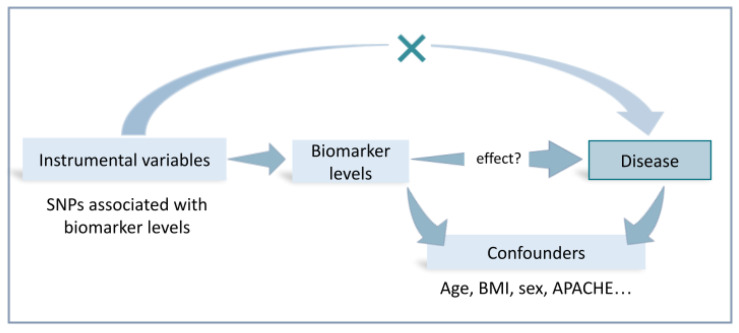
Simplified scheme of a Mendelian randomization (MR) study to determine if a biomarker (e.g., the levels of a plasma protein) is causal of a disease using the genetic variants associated with the biomarkers as instrumental variables. Abbreviations: APACHE: Acute Physiology and Chronic Health Evaluation; BMI: body mass index; SNP: single nucleotide polymorphism.

Statistical genetics approaches such as **Mendelian Randomization** (MR) can be instrumental in determining the causal role of biomarkers. This approach is equivalent to epidemiological randomization studies and makes it possible to determine whether there is a causal effect of a measure of interest, also known as the exposure (e.g., the transcription levels of a gene, levels of a metabolite, a serum biomarker, etc.), in the disease. MR models use the genetic variants associated with the exposure as instrumental variables to infer the causal effect of the exposure. This is based on the premise that individuals randomly inherit alleles. For the models to be robust, these genetic variants must be associated with the disease process only through the exposure [72]. Usually, the genetic variants associated with the exposures are selected from existing GWAS in studies that have aimed to determine their statistical association (Figure 2).

To date, MR has been used in a few studies with critical or respiratory patients, including in sepsis [73], severe COVID-19 [74], idiopathic pulmonary fibrosis [75], or tuberculosis [76], among others. The approach was used to determine the causal effect of the ANG-2 circulating protein levels based on a model of five variants of the *ANGPT2* gene in the development of sepsis-induced ARDS in patients of European ancestry [77]. Similarly, the causal link between the soluble form of RAGE in plasma in European and African American populations was revealed based on a model that contained genetic determinants selected from genome-wide information [78]. MR could also benefit from assessing and/or integrating a larger number of biomarkers or omics data. Following this idea, Dong and colleagues integrated plasma proteomic data in MR studies to determine causal associations with mortality among ARDS patients. In that study, plasma IGFBP7 protein levels were the cause of increased mortality at 28 days, and the effect was mediated by platelet count [79]. Previously, upregulation of the IGFBP7 circulating levels was associated with sepsis-induced lung injury in mouse models [80] and with acute exacerbations in patients with chronic obstructive pulmonary disease [81].

### Genetics of Biomarkers and PRS

Understanding the genetic factors involved in biomarker levels also provides a tool in itself to better understand disease risks. Sinnott-Armstrong and colleagues screened 35 serum and urine protein biomarkers in biobanked samples to find genetic variants associated with their levels, relying on a traditional GWAS approach [82]. Subsequently, to evaluate the prediction of particular traits and diseases from genetic findings, they built and compared PRS models both for the GWAS-derived variants for each biomarker treated individually as well as for the combination of these variants from multiple biomarkers, i.e., deriving a multi-PRS. In that study, multi-PRS improved the prediction of type 2 diabetes, gout, alcoholic cirrhosis, and chronic kidney disease compared to PRS models built only with the variants from individual GWAS. The improvement in multi-PRS models compared to the traditional PRS models is supported by other studies [83,84]. Although multi-PRS models have not been used in ARDS, their application in the diverse biomarkers proposed to date has obvious implications for the better prediction of susceptibility or for stratifying patient prognosis. Conversely, this approach will help to identify novel genetic variants associated with ARDS susceptibility and prognosis.

These findings strongly support that GWAS and other complementary approaches are useful for identifying common risk variants of ARDS susceptibility and for defining which biomarkers could be the most effective in clinical practice. However, most of the rare allele frequency variation remains undetectable with these methods, and thus, complementary approaches are needed to understand its role in ARDS.

## 6. Beyond Common Genetic Variation

### 6.1. Rare Genetic Variation in Disease: Importance, Detection, and Association Testing

The classical SNP array-based GWAS design allows researchers to assess the association of common variation with the trait of interest. However, it is well known that there is an enrichment of pathogenic variants in the rare frequency spectra of genetic variants, and these allow us to explain a higher proportion of disease variability, especially if they are predicted to be loss-of-function variants [85]. Rare variants can affect the phenotype individually or, most commonly, as a combination of alleles. They can even affect one or more genes that could be functionally linked through biological processes [86]. To retain statistical significance, several gene-burden tests and other alternative statistical methods that analyze the variants jointly have been developed to aggregate the effect from different rare variants in a single test [87]. In these situations, standard statistical genetic techniques, such as those typically used for traditional GWAS relying on individual variant tests, do not hold enough statistical power to detect rare variant associations [86,88].

**Next-Generation Sequencing** (NGS) techniques such as Whole-Genome Sequencing (WGS) and Whole-Exome Sequencing (WES) are currently the optimal approximations to study the impact of rare genetic variation in disease risk [89]. With WGS, the entire spectra of genetic variation can be theoretically accessed, irrespective of the location in the genome. Despite that, a strong bias still exists toward the analysis of small genetic variants due to the technical complexities involved in precisely detecting and testing the association of other types of variation [90]. WES has increasingly become a cost-efficient approach, as it solely targets the exons across nearly all known human genes working with the premise that variation in the coding regions might hold more clinical relevance [91].

NGS offers the opportunity to accurately detect new variants, reveal disease-associated genes, and provide a diagnosis. In families, sequencing may reveal inheritance patterns of pathogenic variants and assist in taking measures to prevent risks. This may have important implications in some clinical settings. In this regard, Gong and colleagues used WES to identify the causes of ARDS in a 4-month-old infant. They successfully detected a mutation in the *CD40LG* gene that could explain immunodeficiency. The family pedigree further confirmed that the mother was a carrier of the variant, and an older brother had previously died prematurely after a severe lung infection and sepsis, although genetic information was not available to confirm that this was due to the reported variant [92]. This example reveals the clinical interest of NGS in diagnostics, and its application in the ICU could lead to improvements in diagnosis and rapid medical decision making [93].

### 6.2. The Impact of Rare Genetic Variation in ARDS

The existing literature using NGS to assess the impact of rare genetic variation in the cause of ARDS is scarce. One of these first WES studies used 96 cases and data from publicly available controls (N = 1092) to identify disease-associated variants in sepsis or pneumonia-derived ARDS patients using a genetic association approach [94]. The study prioritized variants for ARDS susceptibility, severity, and 60-day mortality at *ARSD*, *XKR3*, and *ZNF335* genes, which were validated in another small cohort of ARDS cases (N = 117). In the literature, these genes have been involved in sphingolipid metabolism, molecular membrane transportation, transcription regulation, and neural progenitor cell proliferation, respectively. However, the functional mechanisms linking these genes with ARDS are still unknown.

Another small NGS study in 76 sepsis patients was performed by Taudien and colleagues [95]. The study focused on the contribution of rare genetic variants to the post-sepsis disease course, although the results were not specifically linked to ARDS. Of note, the study found an enrichment of rare deleterious variants in genes related to cell signaling and innate immunity pathways that were associated with favorable progression, suggesting a protective role of this type of variation in the immune response to pathogens. These findings are in agreement with a subsequent study by Xu and colleagues, which also focused on the accumulation of non-synonymous genetic variants from WES data [96]. They found that the burden of non-synonymous variants was lower among patients with severe ARDS, ARDS and sepsis, and ARDS with septic shock when compared against non-severe ARDS, ARDS without sepsis, and ARDS without septic shock, respectively. Additionally, the pathway enrichment analysis based on the deleterious variants indicated that the “Extracellular matrix (ECM)-receptor interaction” pathway was significant [96]. This pathway is known to interact with the “Focal adhesion” pathway, where *CDC42* and *HER2* (also known as *ERBB2*) are involved. An enrichment of deleterious variants in these genes was previously associated with a protective role of post-sepsis progression by Taudien et al. [95], while the “Focal adhesion” pathway has been found to be of interest for restoring cell barrier functionality in acute lung injury murine models [97]. Worth noting, *MYLK* is another converging gene of this pathway since this gene encodes the myosin light chain kinase, a central regulator of cell–cell adhesion, extracellular matrix regulation, and vascular integrity and permeability. *MYLK* was prioritized in another WES study [98] of 88 ARDS patients taken from the extremes of the distribution of ventilator-free days. In fact, common *MYLK* variants were previously associated with the risk of trauma-derived ARDS in earlier candidate gene association studies [99].

Additional studies leveraging the power of NGS and with larger sample sizes should be pursued to identify rare risk variants of ARDS. Further research is still needed to interpret the findings and place them in specific pathways to better understand the mechanics of disease pathogenesis [100].

### 6.3. Lessons from the Inborn Errors of Immunity and COVID-19

The **inborn errors of immunity** (IEI) are known to underlie autoimmunity and auto-inflammatory diseases, as well as the increased susceptibility to bacterial or viral infections, such as measles, tuberculosis, Epstein–Barr virus, or influenza virus, among many others [101]. The associated genetic defects inevitably lead to sub-optimal host defense and an increased risk of severe outcomes [102]. A total of 485 different IEIs have been described so far, for which most of the affected genes are related to innate and adaptive host defense pathways [103] and are typically caused by rare damaging variants at single genes. WES studies in IEIs have been most typically conducted in children, probably because of the early onset of critical post-infection symptoms. Many of these IEIs have not yet been associated with severe outcomes such as ARDS, suggesting that penetrance could be incomplete due to low exposure to pathogens or other unknown related mechanisms [104]. However, because of the effects of these damaging variants in response to pathogens and inflammation, the genetic architecture and disease mechanisms underlying IEIs could aid in understanding other critical conditions such as ARDS.

Some IEI examples from the recent literature linked to ARDS have detected an autosomal dominant TLR3 deficiency detected in three unrelated infants with critical influenza-derived ARDS [105]. TLR3 plays a pivotal role in viral infections, recognizes viral replication intermediates, and is involved in the regulation of IFN-α/β expression [106]. Murray and colleagues detected an upregulation of TLR3 in airway epithelial cells isolated from patients with ARDS, and in vitro assays showed that hyperoxic conditions stimulated its expression. Mouse models suggest that anti-TLR3 therapies may have a protective effect on lung damage [107]. Another study identified two inherited compound heterozygotic null variants in *IRF7* in a child who suffered life-threatening ARDS from an influenza A virus infection [108]. This gene encodes a transcription factor, the IFN regulatory factor 7, and transcriptomic studies in patients with ARDS prioritized it as a gene of interest in sepsis-induced ARDS [109]. Hernandez and colleagues also identified a loss-of-function *IRF9* allele in a child that was hospitalized with life-threatening influenza ARDS [110]. This gene plays a critical role in the pathophysiology of ARDS, encoding a transcription factor of many genes involved in the syndrome [111].

These studies based on IEI show how one or a few variants in immune response-related genes can have a major impact on host defense, and also show evidence of the importance that IEIs can have in the development of ARDS due to a dysregulated immune response. Surprisingly, there is an overlap of a total of 22 genes (Table 2) between those listed as an IEI in the last update of the International Union of Immunological Societies Expert Committee [112] and the 238 genes of the ARDS-DB prioritized by intensive manual curation efforts combined with machine learning algorithms [113].

In support of the idea that pathogenic mechanisms of IEIs can help to improve our understanding of ARDS pathogenesis, there has been an exponential increase in studies and a constant reinforcement in the common and different clinical characteristics between COVID-19 ARDS and non-COVID-19 ARDS [119,120]. There is accumulated evidence, stemming mainly from the studies of the COVID Human Genetic Effort at https://www.COVIDhge.com (accessed on 17 February 2023), supporting the role of rare genetic variants in the causes of severe outcomes in COVID-19 critical pneumonia. By relying on WES or WGS studies, seminal studies have reported an enrichment of loss-of-function variants in the type I IFN response genes *TLR3, TRIF, UNC93B1, TBK1, IRF3, IRF7, IFNAR1,* and *IFNAR2* explaining 3.5% of the life-threatening cases [121]. Subsequent studies have replicated or extended these initial results [122,123,124]. In addition, in an elegant study linking the findings in unrelated patients with the inheritance of the identified variants in family-based studies, highly penetrant rare deleterious variants in the X-linked *TLR7* gene were also found to cause critical COVID-19 pneumonia among males [125], supporting the observations of early studies [126,127].

In nearly three years of the COVID-19 pandemic, the balance has tilted towards COVID-induced ARDS [128], where it has become a priority. Based on the robust results provided by the (partial) overlap of findings from GWAS and WES/WGS efforts in large studies to identify the genetic risk of severe COVID-19, it is only a matter of time before similar approximations are applied in non-COVID-19 ARDS. Reuniting knowledge from these sides offers an opportunity to improve the understanding of these conditions, potentially leading to discoveries with implications for novel treatments.

## 7. Genetics and the Treatment Response in ARDS

Genetic variation can also contribute to understanding the individual responses to the treatments of a particular disease. Advances in the understanding of how genetics determines drug responses are central to the development of Precision Medicine, since such information can be used to provide dose-adjustment recommendations and optimize drug therapies based on **pharmacogenetics** [129]. This idea is another frontier to be explored in critically ill patients, although the studies conducted so far involve a small number of patients [130].

For ARDS, there is not yet a specific pharmacological treatment, and the current patient management is based on supportive therapies, such as lung protective ventilation, prone position, and corticosteroids. In this scenario, and despite their use, an open debate remains; the use of corticoids has been shown to improve survival among ARDS patients, including those affected by COVID-19 [10,131,132].

While a number of studies have identified genetic variants associated with corticosteroid treatment responses in respiratory diseases [133,134,135,136], there is a lack of pharmacogenetic studies in ARDS. One study was conducted recently by Jalkanen and colleagues to better understand the harmful effects of administering glucocorticoids together with IFN-β [137,138]. The study is a targeted analysis of the genes encoding the IFN-α/β receptor (*IFNAR1* and *IFNAR2*) with a focus on the common variant rs9984273. The study found that the minor allele (C) and glucocorticoid use were associated with higher 28-day survival compared to the reference allele (T). The study suggested that the use of the two drugs should be avoided in homozygous T allele patients. In addition, the same variant was associated with severe COVID-19 and ARDS risk, which strengthens the potential of studies of genetic overlap between diseases for pharmacological repositioning. While this is in an early stage, further research will be needed involving larger study samples and holistic genomic approaches that may help further disentangle the complexity underlying response regulation [139] and to allow treatment and dosage to be tailored to each patient, rather than applying a one-size-fits-all approach [140,141].

## 8. Knowledge Gaps, Limitations, and Future Prospects

Although genetic studies have improved our understanding of ARDS, there is still limited knowledge of genetic factors involved in the predisposition and severity. This is partly determined by the heterogeneity of the triggers and the multitude of biological processes involved.

In order to improve our understanding of ARDS, some studies have proposed improving the classification of patients into more homogeneous groups sharing clinical or molecular characteristics (i.e., subphenotypes), such as gene expression profiles or other molecular biomarkers [142,143], as it will better reflect differences in prognosis to tailor effective treatments [141,144]. Over the years, several patient classification algorithms have been proposed based on the inflammatory response, the degree of hypoxia, or the origin of the injury (pulmonary or extrapulmonary) [1,141,145]. In this context, ARDS genetic risk factors could show different effects on the different subphenotypes. If so, genetic studies would benefit from the better classification of patients since group-based analyses will help to control the disease heterogeneity in the analyses [146]. Thus, the identification of ARDS endotypes or subphenotypes could represent a breakthrough for Personalized Medicine and for genetic studies. Omic approaches, such as genomics, transcriptomics, or metabolomics, among others, hold the promise to allow identifying such biomarkers for better patient characterization [147,148]. However, key findings must overcome the translational gap between research and its successful translation to clinical use to be able to improve ARDS management and patient outcomes [149].

Another limiting factor is the underrepresentation of certain population groups in the studies. In fact, different studies support that genetic ancestry might underlie genetic susceptibility to the bad prognosis of infections [150,151]. In addition, the SNP heritability of ARDS was estimated to be higher in European versus African American cohorts [31]. Therefore, all the efforts to increase ancestry representation in clinical studies will improve the chances of identifying novel disease loci. An example of the importance of considering ancestry in genetic studies can be found in the study by Pereira and colleagues, who identified a novel severe COVID-19 locus near the *DSTYK* gene. The association comprised a haplotype that was associated with COVID-19 severity only in Brazilian individuals with large European genetic ancestry, suggesting that the latter was the source of the causal variant [152]. However, despite the efforts to increase the study representation of the different genetic ancestries, most genetic studies to date have been conducted on European ancestry populations. The GWAS Diversity Monitor data reflect that more than 90% of the subjects included in the published GWAS are of European ancestry (according to https://gwasdiversitymonitor.com; accessed on 20 February 2023). This situation is paralleled in ARDS [24,31,153]. This lack of diversity in genetic studies limits our understanding of disease and restricts the transferability of findings across populations. For example, disease predictions of PRS models are typically poorer when extrapolated to non-European populations [154,155].

One important factor hampering the genetic studies of ARDS is the heterogeneous clinical practice. This is because current treatment options can be modified by several socioeconomic factors, such as access to technological and economic resources in the hospital, the changing costs of critical care, the changing trends in patient management [156], and the changing definition of ARDS [1]. Along these lines, the American Thoracic Society recommends that, in order to build future equal access to Personalized Medicine for ARDS patients [157], we should ensure that precision medicine advances are available independently of the geographical situation or the resource settings, and of the racial, ethnic, socioeconomic, or demographic differences among patients.

In summary, this review highlights how genetic studies have contributed to the understanding of ARDS so far, from the studies relying on the screening of common variations to alternative approximations focused on testing rare genetic variations. In the last decade, the large impact of GWAS in trait and complex disease research studies has allowed the development of complementary strategies, such as PRS modeling, that can be leveraged to predict and stratify disease risk and assess genetic overlap between traits. The encouraged detection of causal biomarkers by MR, the use of other approaches leveraging multi-trait studies, and studies involving larger and genetically diverse cohorts anticipate promising advances to better understand and treat ARDS.

## Figures and Tables

**Table 1:** Main concept definitions.

Concept	Definition
Variant imputation	Statistical inference of unobserved genotypes in a sample based on the linkage disequilibrium information from genomic reference panels. It is a fundamental procedure in genome-wide association studies to increase the number of variants that can be analyzed in the study, also increasing harmonization of the genetic variants that could be compared across studies.
Genome-wide association studies (GWAS)	A genetic study that compares hundreds of thousands of common imputed variants between cases and controls across the genome to identify those that are more significantly associated with the condition. They are commonly performed assuming additive inheritance models. GWAS findings require extensive replication and functional validation to ensure study robustness.
Polygenic risk score (PRS)	An individual estimate of genetic risk that aggregates the effect of variants previously associated with the disease. They are usually derived from GWAS data and hold promising clinical interest.
Multi-trait analysis	A statistical approach that allows combining the results from different GWAS from correlated traits/diseases to increase the statistical power and detect pleiotropic variants, even from samples overlapping across the studies.
Personalized Medicine	A field that aims to achieve improved diagnosis and individualized clinical treatments based on the genetic or molecular basis of each patient.
Biomarkers	Measurable or observable biological elements that could be used as indicators of a disease or its prognosis.
Mendelian Randomization (MR)	A statistical approach that uses genotypes to assess the causality of an exposure, such as the concentration of a specific biomarker, in a disease or outcome. It uses genotypes as an instrumental variable that are uniquely associated with the disease through exposure.
Next-Generation Sequencing (NGS)	A DNA sequencing technology that enables simultaneous identification of genetic variation across all or parts of the genome using massively parallel sequencing approaches. Whole exome sequencing (WES) targets the coding regions, and whole genome sequencing (WGS) the entire genome.
Inborn errors of immunity (IEI)	Refers to congenital defects that usually affect genes or biological pathways critically involved in immunity. They are usually caused by low-frequency variants leading to patients with a dysregulated immune response and recurrent infections.
Pharmacogenetics	The study of the effects of genetic variation of a patient on the treatment response. Its application in research studies has the potential to improve precision medicine through the search for the most appropriate treatments for each patient.

**Table 2:** Genes causing inborn errors of immunity that are prioritized for their role in ARDS pathogenesis.

Genes	Disease	Inheritance	Associated Features	Reference
*ADA*	Adenosine Deaminase deficiency	AR	Low Natural Killer cell count; bone defects; probable pulmonary alveolar proteinosis; and cognitive defects	[21]
*BRCA1*	Fanconi Anemia Type S	AR	Normal to low Natural Killer cell count; Central Nervous System, skeletal, skin, cardiac, gastrointestinal, or urogenital anomalies; and increased chromosomal breakage	[114]
*CD28*	Cluster Differentiation 28 deficiency	AR	Human papillomavirus susceptibility	[115]
*CXCR2*	C-X-C Motif Chemokine Receptor 2 deficiency	AR	Myelokathexis; recurrent gingivitis; oral ulcers; and hypergammaglobulinemia	[21]
*CXCR4*	Warts, Hypogammaglobulinemia, Infections, and Myelokathexis syndrome (GOF)	AD	Warts (Human papillomavirus) infection; neutropenia; low B-cell count; and hypogammaglobulinemia	[115]
*HMOX1*	Isolated congenital asplenia due to Heme Oxygenase 1 deficiency	AR	Hemolysis; nephritis; and inflammation	[115]
*IL10*	Interleukin 10 deficiency	AR	Inflammatory Bowel Disease folliculitis; recurrent respiratory diseases; and arthritis	[12,115]
*IL1RN*	Deficiency of interleukin-1 receptor antagonist	AR	Neonatal onset of sterile multifocal osteomyelitis; periostitis; and pustulosis	[115]
*ISG15*	ISG15 Ubiquitin-Like Modifier deficiency	AR	Susceptibility to mycobacteria; brain calcifications	[21]
*ITGB2*	Leukocyte Adhesion Deficiency type 1	AR	Delayed cord separation; skin ulcers; periodontitis; and leukocytosis	[115]
*MYD88*	MYD88 Innate Immune Signal Transduction Adaptor deficiency	AR	Bacterial infections (*Streptococcus pyogenes*)	[116]
*NFE2L2*	NFE2-Like BZIP Transcription Factor 2 GOF	AD	Recurrent respiratory and skin infections; growth retardation; developmental delay; white matter cerebral lesions; increased homocysteine levels; and increased expression of stress response genes	[115]
*NFKB1*	Nuclear Factor Kappa B Subunit 1 deficiency	AD	Recurrent sinopulmonary infections; Chronic obstructive pulmonary disease; Epstein–Barr virus proliferation; autoimmune cytopenia; alopecia; and autoimmune thyroiditis	[115]
*NFKBIA*	Anhidrotic ectodermal dysplasia with immune deficiency due to NFKB Inhibitor Alpha GOF	AD	Anhidrotic ectodermal dysplasia; various infections (bacteria, mycobacteria, viruses, and fungi); colitis; variable skin, hair, and teeth defects; and T-cell and monocytic dysfunction	[115]
*STAT1*	Signal Transducer Additionally, Activator of Transcription 1 deficiency (LOF)	AR	Severe viral infections; mycobacterial infections	[117]
	Signal Transducer Additionally, Activator of Transcription 1 GOF	AD	Chronic mucocutaneous candidiasis; fungal, bacterial, and viral (herpes simplex virus) infections; auto-immunity (thyroiditis; diabetes; and cytopenia); and enteropathy	[117]
	Signal Transducer Additionally, Activator of Transcription 1 deficiency (LOF)	AD	Susceptibility to mycobacteria and *Salmonella* spp. infections	[117]
	Signal Transducer Additionally, Activator of Transcription 1 deficiency (LOF)	AR	Severe viral infections; mycobacterial infections	[117]
*STAT3*	Signal Transducer Additionally, Activator of Transcription 3 GOF	AD	Lymphoproliferation; solid organ autoimmunity; recurrent infections	[114]
	Autosomal dominant hyper-IgE syndrome (Job syndrome, due to LOF)	AD	Distinctive facial features (broad nasal bridge); bacterial infections (boils and pulmonary abscesses; pneumatoceles) due to *Staphylococcus aureus*; pulmonary aspergillosis; *Pneumocystis jirovecii* pneumonia; eczema; mucocutaneous candidiasis; hyperextensible joints; osteoporosis and bone fracture; scoliosis; retained primary teeth; and coronary and cerebral aneurysm formation	[114]
*STXBP2*	Familial hemophagocytic lymphohistiocytosis type 5 due to Syntaxin Binding Protein 2/Munc18-2 deficiency	AR or AD	Fever; Hepatosplenomegaly; Childhood Hemophagocytic Lymphohistiocytosis; cytopenia; and enteropathy	[117]
*TIRAP*	TIR Domain Containing Adaptor Protein deficiency	AR	Childhood Staphylococcal disease	[115]
*TNFRSF11A*	TNF Receptor Superfamily Member 11a deficiency associated osteopetrosis	AR	Osteopetrosis	[21]
*TNFRSF6 (FAS)* *	Autoimmune Lymphoproliferative Syndrome due to Fas Cell Surface Death Receptor deficiency	AR or AD	Splenomegaly; adenopathy; autoimmune cytopenia; increased lymphoma risk; IgG and IgA normal or increased levels; elevated serum levels of FasLG protein and Interleukin 10; and increased vitamin B12 levels	[115]
*TNFSF6 (FASLG/FASL)* *	Autoimmune Lymphoproliferative Syndrome due to Fas Ligand deficiency	AR	Splenomegaly; adenopathy; autoimmune cytopenia; Systemic lupus erythematosus; and soluble FasL normal levels	[115]
*USP18*	Ubiquitin Specific Peptidase 18 deficiency	AR	TORCH-like syndrome	[118]

* The currently accepted gene name is indicated in the parenthesis. Abbreviations: AR, autosomal recessive; AD, autosomal dominant; LOF, loss of function; and GOF, gain of function.

## Data Availability

No new data were created or analyzed in this study. Data sharing is not applicable to this article.
